# Ideal time and self-reported time to ejaculate, frequent use of virtual pornography, and disorders of ejaculation among internet users in the Metropolitan Region of São Paulo, Brazil. Cross-sectional study

**DOI:** 10.31744/einstein_journal/2025AO1282

**Published:** 2025-02-26

**Authors:** Margareth de Mello Ferreira dos Reis, Eduardo Augusto Corrêa Barros, Leonardo Monteiro, Cristiano Linck Pazeto, Willy Baccaglini, Sidney Glina

**Affiliations:** 1 Urology Department Centro Universitário FMABC Santo André SP Brazil Urology Department, Centro Universitário FMABC, Santo André, SP, Brazil.

**Keywords:** Erotica, Ejaculation, Premature ejaculation, Diagnostic self evaluation, Sexual behavior, Sexual health, Surveys and questionnaires

## Abstract

**Objective:**

To investigate the association between frequent use of virtual pornography, perceptions of the ideal time to ejaculate, and changes in ejaculation.

**Methods:**

Adult men living in São Paulo metropolis responded to an online anonymous survey between May 2019 and March 2020. The questions were about sociodemographics, sexual behavior, frequency and time of virtual pornography use, and responses to three instruments for assessing ejaculation disorders: self-reported estimated time from vaginal or anal penetration to ejaculation; self-assessment of what is a normal, premature, or delayed ejaculation, and the Premature Ejaculation Diagnostic Tool (PEDT).

**Results:**

Of 528 participants, 27.3% said the ideal time (for them and for the partner) to ejaculate during sex with a partner would be 6-15 minutes, and for 44.1%, longer than 15 minutes. However, 45.8% said they usually ejaculate earlier than the ideal time. Only 55 men (10.4%; 95% confidence interval, 95%CI= 7.9-13.3) participants were frequent users of virtual pornography (defined as the use during solitary masturbation for at least one hour/day every day or many hours/day two to four times/week). Frequent use of pornography was associated with not being heterosexual (odds ratio, OR= 1.81; 95%CI= 1.02-3.23), lower educational level (OR= 2.21; 95%CI= 1.12-4.32) and using virtual pornography during sexual intercourse (OR= 2.58; 95%CI= 1.21-5.48). No associations were observed between frequent virtual pornography use and ejaculation disorders.

**Conclusion:**

Approximately half of the men surveyed are not satisfied with the time they take to ejaculate and around 10% of them use virtual pornography frequently, although this is not associated with ejaculation dysfunction.

## In Brief

Almost half (46%) of adult men living in São Paulo metropolis who responded an anonymous survey think they ejaculate earlier than it would be ideal. Only 10% watched virtual pornography frequently (every day or for several hours 2-4 days a week). No associations were found between ejaculation disorders and frequent use of virtual pornography.

## Highlights

■ About one in every 10 social network male users in São Paulo frequently use virtual pornography.

■ Over half of the respondents believed that the ideal time to ejaculate would be of 15 minutes or more.

■ Frequent use of virtual pornography was not associated with ejaculation disorders.

## Graphical abstract

**Figure:**
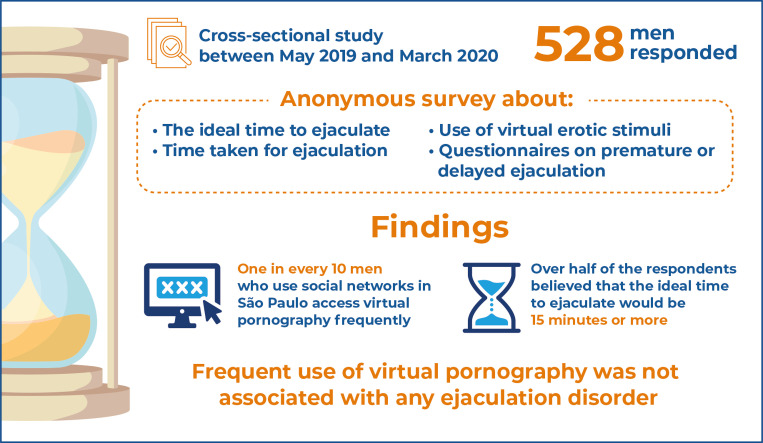

## INTRODUCTION

Male sexual dysfunctions related to orgasm include the absence of control over ejaculation causing suffering and changes in the time for ejaculation that happen in most sexual relationships with a partner for at least six months.^1^ Ejaculation is considered premature when it happens in up to one minute after penetration in a patient presenting the dysfunction during his lifetime or three minutes in cases of acquired premature ejaculation.^2^ Retarded ejaculation happens when there is a significant delay, a low frequency, or absence of ejaculation.^1^ Some authors, trying to make the definition more objective, propose that retarded ejaculation be defined by a delay of 20-25 minutes or more with negative consequences such as distress or concern.^3,4^

The scientific literature on premature ejaculation is much more extensive than that on delayed ejaculation. To investigate the prevalence of premature ejaculation, standardized questionnaires and/or the registration of the time between penetration and ejaculation (measured with a stopwatch or estimated by the man or by the partner) are used. Studies that compared the time between penetration and ejaculation estimated by men and measured with a stopwatch by partners showed that men tend to overestimate the measurement, regardless of whether they present premature ejaculation or not.^5^ However, there is little information about the time that men consider “ideal” to ejaculate for themselves and their partners.

A few recent studies proposed that excessive use of solitary masturbation could explain retarded ejaculation. During masturbation, men can use peculiar movements, hand pressure, and intensity control that are impossible to reproduce during sexual intercourse with penetration or the partner stimulation using the mouth or the hand.^4^ In men with retarded ejaculation, the masturbation can be stimulated by unconventional sexual fantasies^3^ and it is frequently associated with the use of pornographic materials.^6^

Humanity’s interest in images and texts displaying or reporting sexual experiences, or erotica, goes back centuries. Although many of these materials are classified as “pornographic” and under this definition are sought or avoided, operational definitions of pornography for the development of research in the health area are recent.^7^ One of the most used definitions of pornography in studies on its use and effects on health was proposed in 2011 by Reid et al. According to the authors, pornographic material is characterized by “(a) creating or eliciting sexual feelings or thoughts and (b) containing explicit images or descriptions of acts involving the genitals (*e.g*., vaginal or anal intercourse, or masturbation)”.^8^ With the emergence and growth of the internet, the availability of pornographic materials began to occur mostly online (virtual pornography).

In 2021, 84.7% of the Brazilian population aged 10 years or over used the internet.^9^ Data published in May 2022 show that the Brazilian population over 18 years of age spent an average of 91 hours per week connected to the internet^10^ and, according to the internet traffic analysis report published by the company Semrush (www.semrush.com), among the 10 most accessed websites in Brazil in 2022, two offered pornographic content: www.xvideos.com, in third position in the ranking, with 880 million visits until July/22, and www.pornhub.com, in seventh position, with approximately 321 million visits.^11^

Studies suggest that men use online pornography more frequently than women^12^ and that the use of online pornography is associated with solitary masturbation.^6,13^ There is controversy in the literature about the point at which the use of virtual pornography becomes a health problem^14^ and about the association between virtual pornography use and the emergence of sexual problems and/or dysfunctions.^15^ Böthe et al.^16^ highlighted the importance of distinguishing between quantity (frequent use) and severity (problematic use) when investigating the association between virtual pornography use and sexual dysfunctions/problems. While frequent use is estimated from the time a person uses online pornography,^17^ problematic use involves assessing the feeling of loss of control and persistent use of online pornography despite financial, legal, occupational or other problems. relationships resulting from this use.^18^

Three profiles of virtual pornography users were described by Bőthe et al:^14^ (a) infrequent and non-problematic use; (b) very frequent and not problematic use, and (c) very frequent and problematic use. The most frequently observed profile was infrequent and not problematic use (approximately 70% of users), followed by very frequent and not problematic use (19% to 29%). The profile of very frequent and problematic use of pornography, corresponding to experiencing suffering and harmful consequences from virtual pornography use, was the rarest (3% to 8%).

Research questions (RQ) are as follows:

RQ1 — Is there a difference between the time considered ideal to ejaculate during sexual intercourse with a partner and the time taken to ejaculate during solitary masturbation and during sexual intercourse?

RQ2 — What are the sociodemographic characteristics and of sexual behavior associated with frequent use of virtual pornography?

RQ3 — Is there an association between frequent use of virtual pornography and changes in ejaculation (premature or delayed ejaculation)?

## OBJECTIVE

To describe and compare the ideal time to ejaculate (for the man and the partner) during sexual intercourse and the estimated time to ejaculate during solitary masturbation and during intercourse; to investigate the sociodemographic characteristics and sexual behavior associated with the frequent use of virtual pornography (defined as the use of virtual pornography during solitary masturbation for at least one hour a day every day or many hours a day two to four times a week); to investigate the association between frequent use of virtual pornography and changes in ejaculation.

## METHODS

This article presents a new statistical analysis of data collected in a previous study on premature ejaculation among adult male Internet users living in the metropolitan region of São Paulo.^19,20^ The study was approved by the Research Ethics Committee of the *Centro Universitário FMABC* (CAAE: 18453119.2.0000.0082; # 3,832,238). We report this study following the reporting guidelines STROBE (Strengthening the Reporting of Observational Studies in Epidemiology) and CHERRIES (The Checklist for Reporting Results of Internet E-Surveys).

We conducted an anonymous, voluntary, free-access electronic survey with a non-probabilistic convenience sample,^21^ in which we included male internet users residing in the metropolitan region of São Paulo aged 18 years or older. We did not use other exclusion criteria (for example, been sexually active in last months) to reach a sample as close as possible of male general population. Data collection took place between May 2019 and March 2020. This article is part of a greater study about ejaculation disorders in Brazilian men.^19,20^As described before, we did not use monetary or other types of incentives to stimulate survey participation.

Recruitment was made via social media, using snowballing, with invitations posted in the institutional pages as well as in the profiles of the authors, who are urologists and sexual medicine specialists who see patients regularly in a sexual health clinic. Some participants accessed the University or researchers’ Facebook pages and others received the link for the questionnaire by WhatsApp. Either way, participants could repost the advertisements or send the link to the questionnaire to friends and acquaintances or, inviting them to participate. The link gave access to the questionnaire in the Google platform and to a consent form.

We did not use randomization of questions in the survey and we did not use adaptive questioning. The mean number of items (questions) per page was 4,7 and the questionnaire had 7 screens, with all questions mandatory. Participants could not check the consistency of answers before submitting the questionnaire, but they could review their answers through a “back” button. We did not use any imputation of data.^19,20^

We could not calculate response rates as we do not have a denominator (how many people have received or assessed the questionnaire). We could not also calculate completion rate because the participant could only submit the questionnaire if he had answered all questions from the first to the last page. We did not store the IP addresses of participants. We did not use techniques to identify multiple entries or to quantify the time taken to complete the questionnaire.

The questionnaire included sociodemographic variables related to sexual behavior and frequency and duration of virtual pornography use during solitary masturbation. The sociodemographic and anthropometric variables, the ones related to affective and sexual behavior and the variables about virtual pornography during solitary masturbation are listed in table 1. We investigated the time between penetration and ejaculation regardless of whether penetration was anal or vaginal and the time between erection and ejaculation during solitary masturbation.

**Table 1:** Sociodemographic, anthropometric, variables related to affective and sexual behavior and use of virtual pornography during solitary masturbation investigated through an online questionnaire among adult men

Variable	Response categories/units
Age	Years
Race or skin color	White or not white
Schooling	Up to incomplete higher education (university) or complete higher or graduate (post-graduate)
Working situation	Works; studies or without occupation
Individual monthly income	No income; up to five; more than five Brazilian minimum wages
Religion	Yes or no
Body weight	Kilograms
Body height	Centimeters
BMI	Kilograms of body weight/squared meters of height
Obesity (BMI ≥ 30 kg/m^2^)	Yes or no
Physical activity	None; once or twice a week; three times a week or more
Current stable relationship	Yes or no
Sexual orientation	Heterosexual or homosexual/bisexual
Perception of the time taken for ejaculating during solitary masturbation	Up to 2 minutes; 3 to 5 minutes; 6 to 15 minutes
Trying to hold ejaculation	Yes; sometimes; no.
Perception of the time in foreplay before penetration	None or a few minutes; about half an hour; one hour or more
Perception of the time between first penetration and ejaculation during sexual intercourse	Up to 5 minutes; 6 to 15 minutes; more than 15 minutes
Perception of the ideal time between first penetration and ejaculation during sexual intercourse	Up to 5 minutes; 6 to 15 minutes; more than 15 minutes
Perception of the ideal time between first penetration and ejaculation during sexual intercourse according to the partner	Up to 5 minutes; 6 to 15 minutes; more than 15 minutes
Perception of the situation where time for ejaculation is less	Vaginal sex; anal sex; oral sex; masturbation
Use of virtual erotic stimuli during relationship with another person	No; sometimes; often
When you are alone and you masturbate, do you use virtual pornographic stimuli?	No; sometimes; always
For how long do you use virtual pornographic stimuli?	Never; a few minutes per day; at least one hour per day; for many hours of the day; almost the whole day
How frequently do you use virtual pornographic stimuli?	Never; once a week; two to three times a week; on alternate days; daily
For how long do you use virtual pornographic stimuli during masturbation until ejaculation?	Up to five minutes; for 6 to 15 minutes; more than 15 minutes

BMI: body mass index.

We investigated the use of virtual pornography during solitary masturbation because studies have shown that this is the situation where it is used most by men.^6^ We considered as frequent users of pornography stimuli those who accessed virtual pornography for at least one hour a day every day or many hours two or three times per week or in alternate days. The cut-off of seven hours or more per week was used to identify participants with frequent use of virtual pornography, as also applied by Sutton et al.^22^

We evaluated ejaculation in three ways: (1) the estimated time from vaginal or anal penetration to ejaculation reported by the participant (estimated latency time, ELT); (2) the participant’s self-assessment of ejaculation as normal, premature or delayed and (3) the Premature Ejaculation Diagnostic Tool (PEDT),^23^ in its version translated into Brazilian Portuguese.^24^ PEDT consists of five questions whose answers are scored on a scale from 0 to 4, and its final score ranges from 0 to 20 points. Scores ≥11 indicate probable premature ejaculation (PE).^25^

We exported the answers to an Excel spreadsheet and then checked the consistency of the data to remove duplicates and exclude questionnaires with inconsistent answers to questions about the use of virtual pornography (for example, if a man responded he did not use virtual pornography and in the next question stated using it for hours). No imputation technique was used because all questions were mandatory.

Here we describe the quantitative variables using measures of central tendency (mean and/or median) and dispersion (standard deviations, SD) and the qualitative variables using absolute frequencies and percentages. We compared the sociodemographic characteristics of the sample with those of men living in São Paulo Metropolitan Region according to data from the Brazilian Geography and Statistics Institute (IBGE - *Instituto Brasileiro de Geografia e Estatística*), responsible for the population census in Brazil.^26- 30^

Using the kappa coefficient,^31^ we evaluated the agreement between four variables: the time the participant thought was ideal for ejaculating; the time that he thought his partner considered ideal; the time to ejaculate in solitary masturbation, and the time to ejaculate since the first penetration and we calculated 95% confidence intervals (95%CI). We analyzed the discordant pairs using the asymptotic symmetry test (or transmission/imbalance test) described by Spielman et al.^32^

We investigated the association between the outcome “frequent use of pornography” and the sociodemographics and variables related to sexual behavior in two stages: univariate analysis, in which the crude odds ratio (OR) and respective 95%CI were calculated, and then the adjusted analysis, in a logistic regression model (backwards), calculating adjusted ORs and respective 95%CI. After the univariate analysis we selected variables with p<0.20 or those that we considered relevant from a theoretical perspective to test in the adjusted analysis. We used the Hosmer-Lemeshow test to assess the model’s goodness of fit.^33^ We assumed a statistical significance level of p<0.05 and performed statistical analysis using the Stata version 13.1.

## RESULTS

A total of 829 men responded to the questionnaire, as reported before, and we excluded 264 men living outside the area targeted in this study, 1 who was not 18 years old, and 36 who responded in contradictory or incomplete way to the questions about the use of virtual pornography (*e.g*., they first declared not to use virtual pornography but in later questions said they used it frequently for several minutes). We thus included 528 participants in the present analysis, aged 18 to 74 years (mean 26.9; SD 10.3).

Table 2 presents the sociodemographic characteristics of the 528 men compared with data from the IBGE. The participants were younger than the general population in the area. They were predominantly white, and had started university (but most had not yet completed). Half of them declared having a religion. Almost 60% said they practiced a physical activity, and only 18.2% were considered obese. Most were in a stable relationship (58.7%, compared to 61.1% in the general population). They declared being heterosexual less frequently (60.2%) than the general population in the area (94.7%).

**Table 2:** Sociodemographic characteristics of the 528 men surveyed compared to according to data about men(17-21)

Sample	n (%)	São Paulo Metropolitan Region %
Age		Age*
Up to 19 years	123 (23.3)	4.1
20 to 29 years	277 (52.5)	21.1
30 to 39 years	66 (12.5)	23.9
40 years or older	62 (11.7)	50.8
Race/ethnicity		Race/ethnicity^*^
White	356 (67.4)	53.3
Other	172 (32.6)	46.7
Education		Education^†^
Up to incomplete university	329 (62.3)	82.3
Complete university	199 (37.7)	17.7
Sexual orientation		Sexual orientation^≠^
Heterosexual	318 (60.2)	94.7
Not heterosexual	210 (39.8)	1.9
No information	___ ___	3.4
Stable relationship		Stable relationship^§^
No	218 (41.3)	38.9
Yes	310 (58.7)	61.1
Religion		Religion^§^
No	257 (48.7)	11.8
Yes	271 (51.3)	88.2
Obesity (BMI ≥ 30 kg/m^2^)		Obesity^£^
No	432 (81.8)	77.8
Yes	96 (18.2)	22.2
Physical activity		Physical activity^£^
No	337 (63.8)	63.8
Yes	191 (36.2)	36.2

* Data from Pesquisa Nacional por Amostra de Domicílios (PNAD) Contínua 2020-2021 - Características gerais dos moradores. Available at: https://www.ibge.gov.br/estatisticas/sociais/populacao/17270-pnad-continua.html?edicao= 34420&t=destaques. Accessed in 21/11/2022; ^†^ Data from Pesquisa Pesquisa Nacional por Amostra de Domicílios (PNAD) Contínua 2019 - Educação. Available at: https://www.ibge.gov.br/estatisticas/sociais/populacao/17270-pnad-continua.html?edicao=28203&t=destaques. Accessed in 21/11/2022; ^≠^ Data from Pesquisa Nacional de Saúde (PNS) - 2019 - Orientação sexual autoidentificada da população adulta. Available at: https://www.ibge.gov.br/estatisticas/sociais/saude/9160-pesquisa-nacional-de-saude.html?edicao=33558&t=destaques. Accessed in 21/11/2022; ^§^ Data from 2010 Brazilian census. available at: https://www.ibge.gov.br. Accessed in 21/11/2022; ^£^ Data from the Surveillance System for Protective and Risk Factors via Telephone Survey (Vigitel) - 2020. Available at http://plataforma.saude.gov.br/vigitel/. Accessed in 02/04/2024.

Table 3 shows the survey responses about sexual behavior. A total of 171 (32.4%) said they masturbated daily, sometimes more than once a day. During this solitary masturbation, 115 men (21.8%) declared they ejaculated in up to 2 minutes; on the other hand, 198 (37.5%) declared they ejaculated 6 to 15 minutes after penetration. Most participants (54.0%) considered that the ideal time to ejaculate after penetration would be of more than 15 minutes, and 267 (50.6%) said this time was ideal for their partners. The proportion of heterosexual men who reported ejaculating after 6 minutes of solitary masturbation was higher (42.8%) than the observed among non-heterosexual men (32.4%; p=0.037). For both heterosexual and non-heterosexual participants, there was no significant difference in the time to ejaculate that was considered ideal for the respondent and for the partner. There was no difference in the actual time taken to ejaculate either.

**Table 3:** Sexual behavior of male internet users living in the metropolitan region of São Paulo; 2019-2020 (n=528)

	n (%)
Frequency of solitary masturbation	
Up to once a month	33 (6.3)
Once or twice a week	132 (25.0)
Three or four times a week	192 (36.4)
Every day	133 (25.2)
More than once a day	38 (7.2)
Perception of time until ejaculation in solitary masturbation	
Up to 2 minutes	115 (21.8)
3 to 5 minutes	209 (39.6)
6 to 15 minutes	139 (26.3)
More than 15 minutes	65 (12.3)
Perception of time between the first penetration and ejaculation in sexual intercourse	
Up to 2 minutes	49 (9.3)
3 to 5 minutes	105 (19.9)
6 to 15 minutes	195 (36.6)
More than 15 minutes	179 (33.9)
Perception of ideal time between first penetration and ejaculation	
Up to 2 minutes	6 (1.1)
3 to 5 minutes	39 (7.4)
6 to 15 minutes	198 (37.5)
More than 15 minutes	285 (54.0)
Perception of the ideal time between the first penetration and ejaculation for the person with whom you have a relationship	
Up to 2 minutes	7 (1.3)
3 to 5 minutes	50 (9.5)
6 to 15 minutes	204 (38.6)
More than 15 minutes	267 (50.6)
Frequency of use of virtual pornography during solitary masturbation	
Never	35 (6.6)
Once a week	86 (16.3)
Two to three times a week	169 (32.0)
Every other day	98 (18.6)
Daily	140 (26.5)
Time of use of virtual pornography during solitary masturbation	
Never	35 (6.6)
A few minutes of the day	386 (73.1)
At least one hour of the day	85 (16.1)
Many hours of the day	17 (3.2)
Almost all day	5 (1.0)
Frequent use of virtual pornography during solitary masturbation	
No	473 (89.6)
Yes	55 (10.4)
Use of virtual pornography in a relationship with another person	
No	278 (52.7)
Sometimes	194 (36.7)
Frequently	56 (10.6)
Time of erotic stimuli (foreplay) before penetration	
Never/a few minutes	225 (42.6)
About half an hour	248 (47.0)
One hour or more	55 (10.4)
Situation where you think the time to ejaculate is shorter	
Vaginal sex	112 (21.2)
Anal sex	91 (17.2)
Oral sex	80 (15.2)
Masturbation	245 (46.4)
Do you try to hold back your ejaculation?	
Never or sometimes	470 (89.0)
Yes, always	58 (11.0)
Premature ejaculation (according to PEDT)	
No	119 (22.5)
Yes	409 (77.5)
How do you rate your ejaculation?	
Normal	355 (67.2)
Premature	98 (18.6)
Retarded	75 (14.2)

PEDT: Premature Ejaculation Diagnostic Tool.

**Figure 1:**
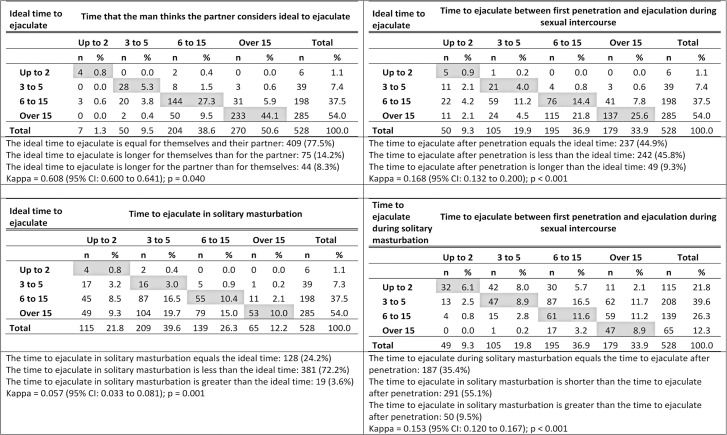
Agreement between the time (in minutes) considered ideal to ejaculate referred by the participant, the time he considers ideal for the partner, the time to ejaculate after the first penetration and the time to ejaculate in solitary masturbation among male internet users living in the metropolitan region from São Paulo; 2019-2020 (n=528) All time measurements in minutes. Gray cells show the concordant answers to the two questions. 95%CI = 95% confidence interval. p corresponds to asymptotic symmetry test.

As shown in figure 1, we observed a substantial agreement between the time men considered ideal to ejaculate and they thought their partners did (kappa = 0.608; 95%CI= 0.600- 0.641); furthermore, a significantly higher proportion of men (14.2%) considered the ideal time to ejaculate longer for themselves than the time they considered ideal for their partnership (8.3%) (p=0.040). However, the agreement was only slight between the time men considered ideal for ejaculating and the time they reported taking to ejaculate both during solitary masturbation (kappa = 0.057; 95%CI= 0.033- 0.081) and after penetration (kappa = 0.168 (95%CI= 0.132- 0.200). In fact, 242 (45.8%) participants reported ejaculating after the first penetration before the time they had declared ideal (p<0.001) and 381 (72.2%) reported ejaculating in solitary masturbation before the time they considered ideal (p<0.001). The agreement between the time to ejaculate in both situations was also slight (kappa = 0.153; 95%CI= 0.120 - 0.167) and 291 (55.1%) reported ejaculating in a shorter time in solitary masturbation than in intercourse with penetration (p<0.001).

Only 35 (6.6%) participants said they did not use virtual pornography during solitary masturbation and most (73.1%) reported using virtual pornography for a few minutes a day. From the combination between frequency and time of pornography use, 55 participants (10.4%; 95%CI= 7.9% - 13.3%) were classified as frequent users of virtual pornography.

Although most men used virtual pornography during solitary masturbation, more than half did not use it during sexual relations with a partner (Table 3). Approximately 40% of men reported not dedicating or dedicating little time to foreplay during sexual intercourse. Masturbation was considered the situation in which the time to ejaculate is shorter by 245 (46.4%) men.

According to ELT, 49 (9.3%) men were classified as having premature ejaculation; 119 (22.5%) had premature ejaculation according to PEDT, and 98 (18.6%) men evaluated their ejaculation as premature ejaculation. Another 75 (14.2%) rated their ejaculation as delayed, and 58 (11.0%) reported always holding back ejaculation.

From the univariate analysis (Table 4), the sociodemographic variables selected for adjustment were: age group, not having completed higher education, obesity, not being heterosexual and not having a stable relationship. Among the variables linked to sexual behavior, the following variables were selected: the perception of time until ejaculation in solitary masturbation, trying to hold back ejaculation, perception of time between first penetration and ejaculation, situation where you think the time to ejaculate is shorter and the use of virtual pornography in relationships with another person. After the adjusted analysis, the characteristics associated with the frequent use of virtual pornography were incomplete higher education, not being heterosexual, and using virtual pornography frequently in relationships with another person (Table 5).

**Table 4:** Univariate analysis of characteristics associated with frequent use of virtual pornography among male internet users residing in the metropolitan region of São Paulo; 2019-2020 (n=528)

	Frequent use of virtual pornography						
	No	Yes		OR_crude_	Inferior limit	Superior limit	p-value*
	n=473	n=55					
	n (%)	n (%)					
Age (years)							0.064
40 or older	59 (95.2)	3 (4.8)		1			
30 to 39	61 (92.4)	5 (7.6)		1.61	0.37	7.05	
20 to 29	250 (90.3)	27 (9.7)		2.12	0.62	7.24	
Up to 19	103 (83.7)	20 (16.3)		3.82	1.15	13.40	
Race/color							0.742
White	320 (89.9)	36 (10.1)		1			
Others	153 (88.9)	19 (11.1)		1.10	0.58	2.05	
Educational level							0.010
Complete higher education or postgraduate	187 (94.0)	12 (6.0)		1			
Incomplete higher education or high school	286 (86.9)	43 (13.1)		2.34	1.17	5.00	
Obesity							0.268
No	390 (90.3)	42 (9.7)		1			
Yes	83 (86.5)	13 (13.5)		1.45	0.68	2.91	
Physical activity							0.791
No	301 (89.3)	36 (10.7)		1			
Yes	172 (90.0)	19 (10.0)		0.92	0.48	1.71	
Sexual orientation							0.038
Heterosexual	292 (91.8)	26 (8.2)		1			
Not-heterosexual	181 (86.2)	29 (13.8)		1.80	1.00	3.28	
Religion							0.729
No	229 (89.1)	28 (10.9)		1			
Yes	244 (90.0)	27 (10.0)		0.90	0.50	1.64	
Stable relationship							0.069
Yes	284 (91.6)	26 (8.4)		1			
No	189 (86.7)	29 (13.3)		1.67	0.92	3.06	
Perception of time until ejaculation in solitary masturbation							0.024
Up to two minutes	103 (89.6)	12 (10.4)		1			
Three to five minutes	196 (93.8)	13 (6.2)		0.57	0.25	1.29	
Six to 15 minutes	121 (87.1)	18 (12.9)		1.28	0.59	2.77	
More than 15 minutes	53 (81.5)	12 (18.5)		1.94	0.82	4.62	
Do you try to hold back your ejaculation?							0.024
No or sometimes	426 (90.6)	44 (9.4)		1			
Yes, always	47 981.0)	11 (19.0)		2.17	1.09	4.83	
Time of erotic stimuli (foreplay) before penetration							0.720
None/few minutes	204 (90.7)	21 (9.3)		1			
About half an hour	221 (89.1)	27 (10.9)		1.19	0.65	2.16	
One hour or more	48 (87.3)	7 (12.7)		1.41	0.57	3.52	
Perception of time between the first penetration and ejaculation in sexual intercourse							0.081
Up to two minutes	45 (91.8)	4 (8.2)		1			
Three to five minutes	95 (90.5)	10 (9.5)		1.18	0.35	3.98	
Six to 15 minutes	181 (92.8)	14 (7.2)		0.81	0.27	2.77	
More than 15 minutes	152 (84.9)	27 (15.1)		2.00	0.66	6.01	
Perception of ideal time between first penetration and ejaculation							0.542
Up to five minutes	39 (86.7)	6 (13.3)		1			
Six to 15 minutes	175 (88.4)	23 (11.6)		0.75	0.33	2.24	
More than 15 minutes	259 (90.9)	26 (9.1)		0.65	0.25	1.69	

*χ^2^ test.

**Table 5:** Final adjusted model (adjusted for all variables present in the model) of characteristics associated with frequent use of virtual pornography among male internet users residing in the metropolitan region of São Paulo participating in the study. 2019-2020 (n=528)

	Frequent use of virtual pornography			
	OR_adjusted_	Inferior limit	Superior limit	p-value*
Educational level				
Complete higher education or postgraduate	1			
Incomplete higher education or high school	2.21	1.12	4.36	0.021
Sexual orientation				
Heterosexual	1			
Not-heterosexual	1.81	1.02	3.23	0.043
Use of virtual pornography in a relationship with another person				
No	1			
Yes, sometimes	0.73	0.38	1.43	0.365
Yes, frequently	2.58	1.21	5.48	0.014

*Wald test. Hosmer-Lemeshow adjustment test: 0.842.

## DISCUSSION

In our study, we observed that 27.3% of men believed that the ideal time to ejaculate for themselves and their partner would be between 6 and 15 minutes and that 44.1% believed that the ideal time for both would be greater than 15 minutes. These high expectations were not met, as 45.8% of men reported ejaculation times after penetration that were lower than what they considered ideal.

Little is known about the time men in the general population consider ideal to ejaculate or the time they actually take to ejaculate. One study did evaluate this issue about the difference between the desired and achieved lag between the start of the intercourse and the climax.^34^ Both heterosexual and non-heterosexual men considered that the ideal time would be 10 minutes — but the actual median time taken to ejaculate was 6 minutes for heterosexual and 5 minutes for non-heterosexual (with no statistically significant difference between them). Heterosexual, homosexual and bisexual men value mechanical aspects of sexual performance (erection and ejaculation)^35^ and this could feed the unrealistic expectations we observed in our study: an ideal time to ejaculate higher than the actual time, independently of sexual orientation.

The actual time taken to ejaculate was referred by 56.8% of the participants in our study as 3 to 15 minutes after penetration. This self-reported latency time is similar to the average found in a study with 474 men conducted in five countries, where the global latency was of 8.5 minutes (SD: 7.5 minutes), varying from 5.2 minutes (SD: 1.7 minutes) in Spain to 11.6 minutes (SD 8.4) in the United Kingdom.^36^ However, studies surveying the general population like these are still scarce, which makes the diagnostic classifications currently available be based on expert opinion only, instead of epidemiological data.^34^ There is clearly a need for more epidemiological studies on the time for ejaculation, especially those considering other sexual practices beyond vaginal penetration.

Such studies would allow the realization of how the use of virtual pornography can affect ejaculation time. In our study, we observed more than 90% of men referring the use of virtual pornography at least once a week. There is a possibility that the much higher expectations about the time to ejaculate than the actual time be due to unrealistic references provided by porn. In pornographic films, actors often use medication and other resources to “boost up” their sexual performance, and the situations portrayed in the films do not correspond to people’s daily lives. The difference between the objectively measured time and the time considered ideal for ejaculating may be even greater, as studies show that men tend to overestimate it after the first penetration compared to measurements taken by their partners.^5^ Furthermore, 11.0% of participants declared they always try to hold back ejaculation, and this may not reflect an attempt to deal with losing control over ejaculation but rather an attempt to achieve an idealized sexual performance.

In our study, 10.4% of men used virtual pornography during solitary masturbation frequently. The results of this type of survey are quite varied in the literature. Preliminary data from the multicenter International Sex Survey,^37^ including participants from 46 countries, shows a proportion of online pornography use once a week of 38% — this proportion was 16.4% in our study. For a frequency of more than six times a week of pornography use, there were also some differences between our data and the International Sex Survey and two other studies, one with US military men and the other with Polish students: 8% in the International Sex Survey;^38^ 9.3%^12^ among US military men (mostly virtual), and 10.7%^26^ among Polish students^39^ — in our study, 26.7% reported using virtual pornography daily.

We observed that the characteristics associated with frequent use of virtual pornography were: not being heterosexual, not having completed higher education, and using virtual pornography frequently in relationships with another person. Bőthe et al.^17^ also observed a higher proportion of frequent use of virtual pornography among non-heterosexual men compared to heterosexual men and homo and heterosexual women. The authors suggested that non-heterosexual men might have more difficulty establishing affective-sexual relationships with stable partners and resort to the use of virtual pornography and a greater number of casual sexual partners; and that the use of virtual pornography could also be a comfortable and accessible form of relief from stress and unpleasant emotions. The association between not being heterosexual and greater use of virtual pornography was also observed in a study that included young Australians aged between 15 and 29 years; the authors suggested that pornographic materials could become important instruments for the sexual education of young people (especially those who are not heterosexual).^40^

In our study, 39.8% declared themselves as not heterosexual. These data are quite different from the 2019 National Health Survey - Self-identified Orientation of the Adult Population^29^ carried out by IBGE, in which only 1.4% of the male population declared themselves to be homosexual and 0.5% bisexual. In the National Survey, the proportion of men who refused to answer about their sexual orientation (2.3%) was higher than the sum of the homo and bisexual populations, and 1.1% of men reported not knowing their sexual orientation. These data suggest the difficulty people have in declaring and/or realizing their sexual orientation in our country. Part of the fear of declaring sexual orientation may be due to the violence to which the LGBT population is exposed: according to the dossier “Deaths and Violence against LGBTI+ in Brazil - 2021”, produced in 2021, 316 violent deaths of LGBTI+ people were recorded, of which 145 were gay men, and about 90% were homicides or robbery.^41^

In our study, the association between younger age and a higher proportion of frequent use of virtual pornography observed in the univariate analysis was not maintained after considering all the variables together, in contrast to the findings of other authors.^18^ Possible explanations for this finding are the age composition of our sample, mainly composed of men aged up to 29 years, but not restricted to university students, as in other existing studies.^39,42^ On the other hand, in our research, education below complete higher education was associated with a greater chance of frequent use of virtual pornography, in disagreement with the findings among young Australians.^40^

We observed that men who were frequent users of virtual pornography during solitary masturbation were significantly more likely to use virtual pornography during sexual intercourse with another person. There is ample debate in the literature about the effects of using virtual pornography on emotional relationships. Studies suggest that the effect on relationships depends on the context in which it occurs: virtual pornography used jointly by partners is associated with higher levels of satisfaction with the relationship,^43^ while solitary use is associated with lower levels of personal satisfaction and sexual behavior among men.^44^

We did not observe significant associations between frequent use of virtual pornography and ejaculation alterations, in agreement with the findings of other studies.^16,45,46^ As observed by Whelan et al, frequent use of virtual pornography is not associated with premature ejaculation, although self-perceived virtual pornography addiction (regardless of the frequency of use) is.^47^ In their study, among men who considered themselves “addicts”, feelings of guilt, shame and anxiety related to moral incongruence could interfere with their sexual function and satisfaction. We did not find an association between religion and the frequent use of virtual pornography either, as observed by Grubbs et al.^48^ However, although having a religion does not seem to change the amount of time spent using virtual pornography, it could still lead to a greater chance of perceiving this use as problematic or as an addiction to pornography.^48,49^

Our study has some limitations. As for any other cross-sectional study, the study design does not allow the establishment of causal relationships — however, the associations found might be relevant for healthcare. The convenience sample used, recruited by the internet and using snowballing, might have brought some self-selection bias.^50^ However, almost all epidemiological studies on sexual dysfunction and other aspects of sexuality are conducted using convenience samples of volunteers recruited in public places, health services, and via internet due to the sensitive nature of the topic. The same applies to premature ejaculation.^19^ The sample in this study was restricted to users of social networks, with a sociodemographic profile that may be different from the general male population of the metropolitan region of São Paulo. This can make it difficult to generalize the results for the population who does not use online social networks.

Another limitation that could be noted is the self-reporting nature of the information, including those necessary for the evaluation of ejaculation, with no clinical verification. We chose to ask about the ideal time to ejaculate and the time to ejaculate during masturbation and penetration using time intervals as response categories (and not measured in minutes or seconds). Although this methodological choice may have led to a loss of statistical power, we believe that it increased the probability of participants answering the questions. However, the anonymity of the questions increases the probability that the participants answered the questions sincerely.

## CONCLUSION

We observed that approximately 1 in every 10 social network users in the metropolitan region of São Paulo frequently use virtual pornography, with no significant associations between frequent use of virtual pornography and ejaculation alterations. Over half of the respondents believed that the ideal time would be of 15 minutes or more. Most men reported ejaculating in less time than the time they considered ideal. Our results call attention to the need for more in-depth research on the pattern of virtual pornography use and on the effects of unrealistic expectations about ejaculation in our environment and its effects on users and their partners.
